# Evaluation on maturity and stability of organic fertilisers in semi-arid Ethiopian Rift Valley

**DOI:** 10.1038/s41598-021-83611-4

**Published:** 2021-02-17

**Authors:** Shiro Mukai, Wataru Oyanagi

**Affiliations:** 1 Independent Scholar, 1-19-7 Omorinishi, Ota-ku, Tokyo, 143-0015 Japan; 2 Niigata Agricultural Research Institute Livestock Research Center, Niigata, 955-0143 Japan

**Keywords:** Ecology, Plant sciences, Environmental sciences

## Abstract

Case studies that have comprehensively examined local organic fertilisers (OFs) for their maturity and stability are rare in sub-Saharan Africa. Farmers in the semi-arid Ethiopian Rift Valley use indigenous compost (*kosi*) and household wastes for OFs. With the entry of fast compost that was introduced by the administration, maturity and stability of these OFs were assessed. Their maturity was assessed by: monitoring pile temperature and volume, pH, organic matter and total nitrogen contents, and carbon to nitrogen ratio; determination of NO_3_^–^ to NH_4_^+^ ratio; and respirometric measurement of CO_2_ evolution. Their stability was assessed by weed seed germination tests and phytotoxicity bioassays. Weed seeds that were originally contained in the feedstock of the *kosi* and fast compost samples became inactive during the composting process. The CO_2_ evolution tests and phytotoxicity bioassays indicated a probable presence of some phytotoxic compounds in the *kosi*. Mature *kosi* and immature *kosi* in a *kosi* pile should be mixed before the field application. Some samples (15%) of the household wastes contained weed seeds. The combination of several assessment methods used in this study and determination methods for nitrogen components using RQ-flex is considered to be effective for on-site quality assessment of OFs in sub-Saharan Africa.

## Introduction

Sub-Saharan Africa (SSA) remains one of the last global regions struggling to attain food security. Even if the current on-farm yields on existing cropland in SSA were raised to the yield potential, they would be inadequate to meet future demand for cereals, which in 2050 is expected to be 335% of the 2010 level^1^. Soil fertility decline due to high agricultural and grazing pressures and soil erosion has been a serious problem in Eastern and Southern Africa^2^, including Ethiopia^3^. Partly because farm-level inorganic fertiliser prices in SSA are among the highest in the world, most farmers are constrained by a shortage of cash to use inorganic fertilisers^4^. In some areas which undergo a serious nutrient loss caused mainly by soil erosion from the crop fields, the number of farmers who use organic fertilisers (OFs) and inorganic fertilisers has been increasing^5–7^. This tendency is in line with the integrated soil fertility management, the combined use of organic and inorganic fertilisers, enhanced in SSA since the 1990s^8^.

Maturity and stability are important parameters for manure/compost quality assessment^9^. A well-accepted definition of compost stability is the rate or degree of organic matter (OM) decomposition. Compost maturity generally refers to the degree of decomposition of phytotoxic organic substances produced during the active composting phase and to the absence of pathogens and visible weed seeds^10^. A low carbon (C):nitrogen (N) ratio (C:N ratio) is considered to be an indicator of compost maturity. Although the final C:N ratio has a wide variety depending on its feedstock, the final C:N ratio for matured compost is said to be 10–15^11^. The mean C:N ratio of cattle manure/compost in Eastern and Southern Africa is as high as 23 (*n* = 506), and that of sheep and goat manure is 22 (*n* = 75)^12^. Easily decomposable OM contained in immature OFs or the OFs with a high C:N ratio is rapidly decomposed in the soil, which may cause damage to crops by depriving plant roots/seeds of oxygen or N^13^. If an oxygen shortage occurs in the decomposition process of easily decomposable OM, anaerobic fermentation prevails, and possibly phytotoxic compounds such as NH_3_^+^ or short-chain organic acids that retard the plant growth are left undecomposed. Many studies suggested cattle manure/compost with a high C:N ratio caused initial N immobilisation shortly after applying to the soil, possibly leading to a lower rate of seedling emergence and yield reduction^14,15^. Because livestock is usually grazed freely in open fields in Eastern and Southern Africa, the application of immature OFs possibly increases the weed biomass and weed species in the field^16^.

Case studies on the quality assessment of local OFs in Eastern and Southern Africa include (1) analyses of different decomposition characteristics and nutrient availability to establish a decision-support system for farmers^17,18^; (2) analyses of how farmers’ management practices affect the chemical composition of manure^19–21^; (3) analysis of the relationship between farmers’ simple methods for manure/compost quality assessment and their chemical compositions^22^; and (4) analyses of how different production and storage methods of cattle manure affected the manure organic N contents and the manure mineral N released in soil^23^. However, case studies that comprehensively examined the maturity and stability of local manure/compost are rare^24^. Strengthening linkages between research, extension services, and farmers is likely to be one of the requirements for sustainable soil fertility management in SSA^25^. Local research institutes closer to farmers are expected to conduct a quality soil testing, establish regionally specific fertiliser response recommendations, recommend improved/updated cropping system to farmers^25^. However, these local research institutes in SSA are often less endowed with an affluent budget and expensive experimental instruments^24^.

The objectives of this study are, first, to assess the maturity and stability of local OFs used in the semi-arid Ethiopian Rift Valley. Considering the repeatability of this study in Eastern and Southern Africa, the second objective is to create a case study on the quality (maturity and stability) assessment of OFs, which can be implemented in agricultural experiment stations that equip a certain level of the experimental facility. Enormous case studies and many review papers on the maturity and stability of OFs have been published by introducing various assessment methods. Among those, this study selected some assessment methods that have certain credibility and do not require expensive measurement instruments. This study also adopted simple determination methods for N components using a spectrometer reading test (Merck RQFlex reflectometer)^12^.

## Materials and methods

### Study area and organic fertilisers tested

**Figure 1:**
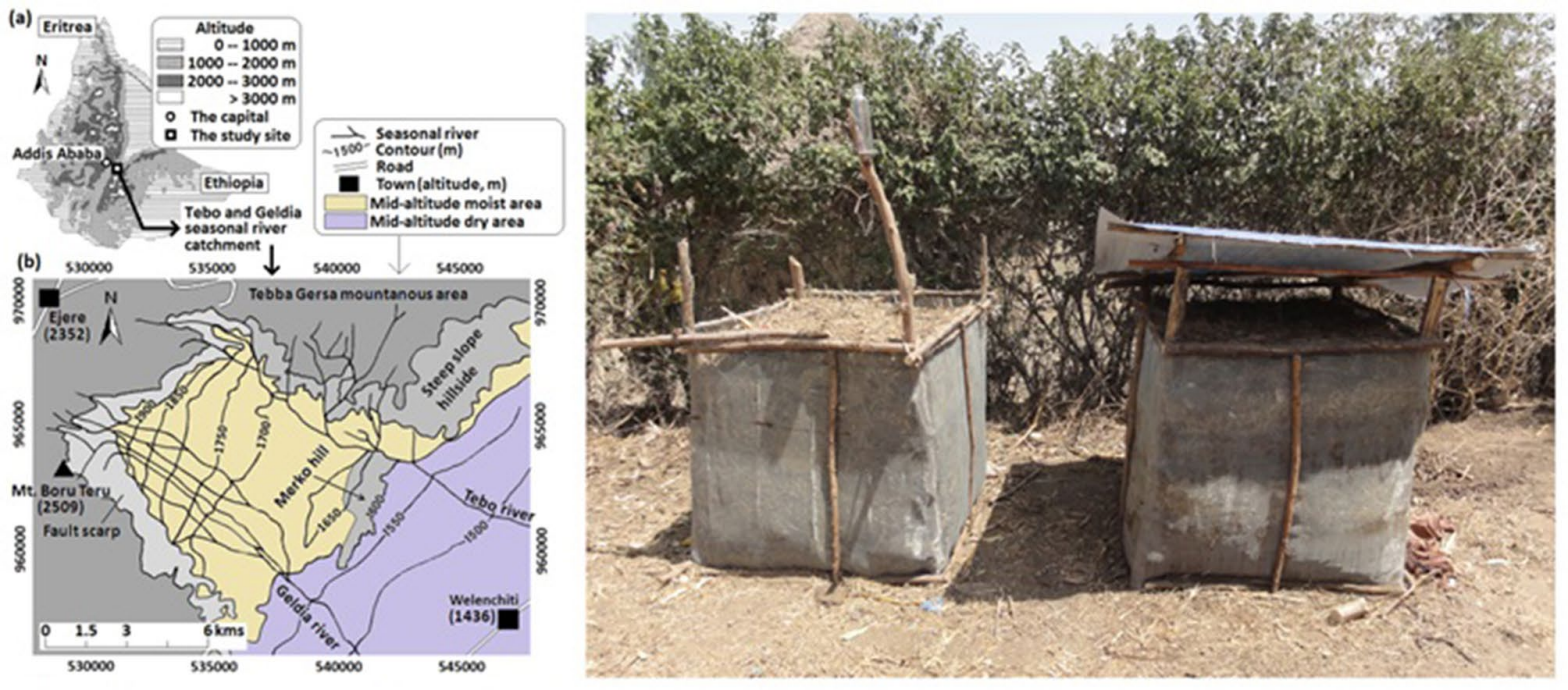
The study area (left figure) and the test-*kosi* pile (left in the right figure) and the fast compost pile (right in the right figure).

Most of the Tebo and Geldia seasonal rivers catchments (the study area; coordinate system, UTM WGS 1984, 528600, 973100 –541700, 951600; Fig. 1) are located in the semi-arid Ethiopian Rift Valley. The catchment areas are categorised into two sub-areas in terms of major maize growing areas in Ethiopia: mid-altitude dry (1000–1600 m a.s.l.; annual rainfall 800–1000 mm) and mid-altitude moist (1600–1800 m a.s.l.; annual rainfall 1000–1250 mm) sub-areas^12^. These two categories cover 63% of the total maize growing area in Ethiopia. The major crops in the mid-altitude dry sub-area of the catchments area are sorghum, tef (*Eragrostis tef*), and maize, whereas those in the mid-altitude moist sub-area are wheat, tef, and maize^12^.

Most households in the semi-arid Ethiopian Rift Valley hold continuously cropped maize fields (locally referred to as *aradas*^26^), which acquire fertility from the regular input of OFs, such as compost (locally, *kosi*) or household wastes^27^. *Kosi* is made from a variety of locally available organic materials, such as various types of animal dung, kitchen ash, crop residue, and feed refusals. These compost materials are piled up in the corners of house-yards for several months to a few years for decomposition^27^. Household wastes are substantially a variety of organic materials themselves that also comprise *kosi*. It is mainly a housewife who collects these organic materials through house-yard sweeping and dumps directly on the *arada* that adjoins the homestead every few days. It has been since the beginning of the 2000s when the district agricultural office began giving fast compost training to farmers^28^.

### Compost maturity and stability tests

Microorganisms break down the chemical bonds of organic materials in the presence of oxygen and moisture, giving off heat. Rynk et al.^29^ found that maintaining a stable temperature of > 62.8 °C within the compost pile, for more than three consecutive days had been effective for the destruction of most human pathogens, insect larvae, and weed seeds within the compost pile. Monitoring the volume of compost pile during the biodegradation process is another physical test to evaluate compost stability^30,31^. Thus, monitoring compost pile temperature (self-heating test) and volume can be used as a simple and rapid method for assessing compost maturity or stability^32^.

The pH values of compost usually increase during the early stage of composting generally to above 8, caused by the release of ammonia, and then decrease slowly but steadily as ammonium (NH_4_^+^) is nitrified to approach neutral values as compost matures^33^. However, numerous studies have demonstrated that pH trends and final values over the composting are highly dependent on feedstock materials^32^.

C:N ratio generally decreases throughout the composting process due to the C losses^13^; however, the wide variability in feedstocks leads to variability in the final C:N ratios in different composts, making it difficult to place an absolute limit on C:N ratio that will be applicable to all feedstocks^32^. The pH values and C:N ratio of compost are useful in compost maturity evaluation if initial and final values are compared and if it is monitored in conjunction with other parameters for compost maturity^32^.

Sánchez-Monedero et al.^34^, which analysed the evolution of the different forms of N during the composting of different feedstocks, found that the greatest concentration of NH_4_^+^ coincided with the most intense period of OM degradation, whereas the highest concentrations of nitrate (NO_3_^−^) were always produced at the end of maturation. They concluded that NO_3_^−^ to NH_4_^+^ ratio is a clear indicator of the compost stability. Wichuk and McCartney^32^ recommended that, because the ratio varied in mature composts, monitoring the ratio several times throughout the different stages of the composting process, rather than relying on the final value alone. NO_3_^−^:NH_4_^+^ ratio can be an effective indicator to evaluate the stability of *kosi*, fast compost, and household wastes, which have the same organic materials in common but are the products being in different phases of the composting process.

Respirometry (CO_2_ evolution rate or O_2_ uptake rate) has been widely used to evaluate the microbial activity and therefore, the stability of a compost sample^9^. The equipment for respirometric assays based on CO_2_ evolution is generally simple and easy to use; however, the main disadvantage of these methods is that they are unable to distinguish between CO_2_ produced aerobically from that produced anaerobically^9^. Respirometric assays based on O_2_ uptake are the most accepted methods for determining the biological activity of material; however, their main disadvantage is that they need more expensive and troublesome instrumentation and more skilled labour^9^. Respirometric assays are not without flaws; nevertheless, many researchers recommended using either of the two respirometric assays or self-heating (in combination with a plant bioassay) to evaluate compost stability^32^.

Gómez-Brandón et al.^13^ compared several parameters and found that the change in dissolved organic carbon (DOC) with composting time gave a good indication of stability. However, the determination of DOC content requires expensive laboratory instruments such as absorption spectrophotometer. Instead, Wu et al.^10^ and Gómez-Brandón et al.^13^ found a significant correlation between DOC and microbial respiration. They referred to the way of evaluating compost stability based on CO_2_ evolution and assessing compost maturity based on phytotoxicity bioassay (seed germination). Compost maturity is generally determined by phytotoxicity bioassay^32^.

To date, no stand-alone method exists to assess compost maturity, mainly because of the wide variety of composting feedstocks and management practices^35^. A more thorough evaluation of both the stability and maturity states of compost could be obtained using a combination of tests^32^. An appropriate field test method would need to be rapid and sufficiently straightforward for operators to use^32^.

Considering these, (1) monitoring pile temperature and volume changes and (2) determinations of pH, OM, total N, and C:N ratio (total organic C to total N ratio) over the composting process; (3) determination of the final NO_3_^−^:NH_4_^+^ ratio; (4) CO_2_ evolution test; and (5) phytotoxicity bioassay were combined to evaluate stability and maturity of the OFs, i.e., *kosi*, fast compost, and household wastes, in this study. Besides, because weed proliferation in *arada* fields is the primary cause of maize yield decline^36^, (6) a weed seed germination test was conducted.

### Monitoring of physical and chemical changes in compost piles

Five farmers who participated in the fast compost training from each of the two sub-areas were requested to make *kosi* and fast compost. The temperatures and volumes of the *kosi* and compost piles were monitored only in the mid-altitude dry sub-area over 90 days from the commencement day when the OF feedstock (organic materials) had been piled up (the *kosi* pile prepared was referred to as “test-*kosi* pile”). This test was conducted once in each 2014 and 2015. To ease the pile volume measurement, the organic materials collected from the farmers’ backyards were piled up in a rectangular wooden frame (1 m in width, 1 m in length, 1.5 m in depth; Fig. 1). Fast compost was made following the technical guidance of MoARD^28^: each 20-cm-deep layer of the (1) maize and sorghum stalks, (2) animal dungs, and (3) tef residue and feed refusals were piled up in turn until it reached the top of the pile. The total depth of each material layer was arranged to be the same between (1), (2), and (3). (4) Ash (0.5 kg m^−2^) was sprinkled over each layer of the (1) and (3). Some humic soil (1–2 cm deep) was spread on top of each layer. Water was regularly added to keep the pile moist. Once every 21 days, all the organic materials in the piles were turned over to mix the materials. This process was repeated to make fast compost ready in 3 months^28^. The same varieties of the organic materials were used for the *kosi* feedstock, but those compositions and proportions were decided by the individual farmer. The fast compost pile had a ceiling so that rainfall did not enter inside, whereas the test-*kosi* pile was rainfed (Fig. 1). Daily rainfall was measured by a simple rain-gauge installed near the test-*kosi* piles.

Farmers in Eastern and Southern Africa carry OFs from their *kraals* (cattle parking lot) and cattle sheds to the field and integrate it into their fields by ploughing operations carried out a couple of weeks later^37^. Farmers in the semi-arid Ethiopian Rift Valley plough maize fields 3–4 times and tef fields 4–5 times before seeding^36^. For both the crops, many farmers integrate the applied OFs into the soil at the ploughing time implemented immediately before the seeding or at the previous ploughing time. For maize, this period corresponds to the beginning of the rainy season from late-May to the beginning of June^36^. As soon as they carry *kosi* to their fields, they begin the next *kosi* making. Thus, the test-*kosi* and fast compost samples were begun to prepare on 4th June in 2014 and 2nd June in 2015.

Daily temperatures were measured at randomly selected five points in the test-*kosi* and fast compost piles by a temperature probe (SINWA digital thermometer H1). Those mean values were designated as the daily temperature. The depths of the piles were measured every 10 days over the monitoring period, which were converted into volume. Similarly, pile pH (HORIBA portable pH meter D-210P) and total N and OM contents (loss-on-ignition method; ignition temperature 500${}^{o}c$, overnight) of the piles were determined.

Only for pH, it was measured at the 2nd day of the monitoring together with the regular measurement made every 10 days, including the 1st day of the monitoring. Total organic C was estimated from the OM content determined^38^, which was used to determine C:N ratio.

Total N in the sample was determined by the following on-site proximate analysis methods^39^ (Table S1 in Supplementary Information online): after 1.0 g (dry matter) of the sample was placed in a 500-mL tall beaker and 8 mL of sulphuric acid was added, 4 mL of 35% hydrogen peroxide was added twice, which was capped with a dish. After a vigorous chemical reaction was settled, the tall beaker was heated for 5 min. After the beaker was cooled down, 2 mL of hydrogen peroxide was added, and then heated for 3 min; this operation was repeated six times. The solution was transferred to a volumetric flask, and water was filled to the marked line of 100 mL. Because Reflectquant ammonium test (0.2–7.0 mg L^−1^ NH_4_^+^) requires a test solution regulated in pH 4–13, after 29 mL of water was added to 1 mL of the solution, 0.4 g of calcium hydroxide was added, which was stirred hard. The filtrate was reacted with a Reflectquant ammonium test, and NH_4_^+^ was determined with an RQFlex in a thermostat bath kept at 30 °C. A standard solution for NH_4_^+^ (3.0 μg mL^−1^) was simultaneously determined to correct determined NH_4_^+^ in the sample. Corrected NH_4_^+^ in the sample (X) was converted to total N (Y) using the equation^39^, Y = 0.830 X (Table S1 in Supplementary Information online).

In the village in the mid-altitude dry sub-area where the sample piles were established, abundant pumice flow deposits were observed in soils; this was so in the humic soil added to the compost pile. The weight of pumice in the samples collected from the test-*kosi* and fast compost piles, if any, was measured, which was deducted from the crude ash mass measured after combustion.

### Weed seeds germination test

A weed germination test was conducted in 2015 as follow: a fast compost sample and test-*kosi* sample were collected from the each of the 5 fast compost and 5 test-*kosi* piles set in the two sub-areas in 90 days of the monitoring period. Thus, 10 fast compost samples and 10 test-*kosi* samples were prepared. Besides, 5 *kosi* and 5 household wastes samples were collected from 5 farmers’ backyards in both the sub-areas (a *kosi* sample collected from farmers’ backyard was referred to as a farmer-*kosi* sample). In collecting a household wastes sample from a farmer’s backyard, approximately a 10 g sample was collected from each of the five places in the backyard, mixed to make it a composite sample. From each of the 10 fast compost, 10 test-*kosi*, 10 farmer-*kosi*, and 10 household wastes samples, 8 samples were collected to prepare 80 fast compost, 80 test-*kosi*, 80 farmer-*kosi*, and 80 household wastes samples. A filter paper was placed on a petri dish 9 cm in diameter, on which a sample was spread. The sample in the petri dish was uniformly watered and placed in the constant temperature room (kept at 25 °C) in Melkassa Agricultural Research Center for 10 days. Species of the plants germinated were identified at the National herbarium of Ethiopia. The 10 fast compost, 10 test-*kosi*, 10 farmer-*kosi*, and 10 household wastes samples tested in the weed germination test were also used for the NO_3_^−^:NH_4_^+^ ratio determination test, CO_2_ evolution test, and phytotoxicity bioassay.

### NO_3_:NH_4_^+^ ratio determination

NH_4_^+^ and NO_3_^−^ in the 10 fast compost samples, 10 test-*kosi* samples, and 10 farmer-*kosi* samples were determined, from which NO_3_^−^:NH_4_^+^ ratios were calculated.

Tanahashi et al.^40^ found that cattle and swine manures contained the fraction of NH_4_^+^ that cannot be extracted by potassium chloride (ammonium magnesium phosphate; MAP). They examined 59 cattle manures (26 dairy, 28 beef, and 5 dairy and beef mix) and 52 swine manures made by various production methods for an appropriate extraction method of NH_4_^+^ containing MAP^40^. As a result, they found that inorganic N containing MAP extracted by 0.5 mol L^−1^ hydrochloric acid in the condition of the 1–10 ratio of dry manure weight (g) and extract volume (mL) had the strongest relationship (R^2^ = 0.851, including some outliers) with inorganic N available in the culture soil used for laboratory incubations (30 °C, 4 weeks). Thus, this study used hydrochloric acid to extract inorganic N from the OFs. After 0.5 mol L^−1^ hydrochloric acid solution (100 mL) was added to 10 g of the sample, the solution was stirred by a mixer for 2 min to make an extract. The extract was diluted, if necessary, and was reacted with Reflectquant ammonium test (measuring range of 0.2–7.0 mg L^−1^ NH_4_^+^) and Reflectquant nitrate test (5–225 mg L^−1^ NO_3_^−^) to determine NH_4_^+^ and NO_3_^−^ contents with an RQFlex, respectively (Table S1 in Supplementary Information online).

### CO_2_ evolution test

**Figure 2:**
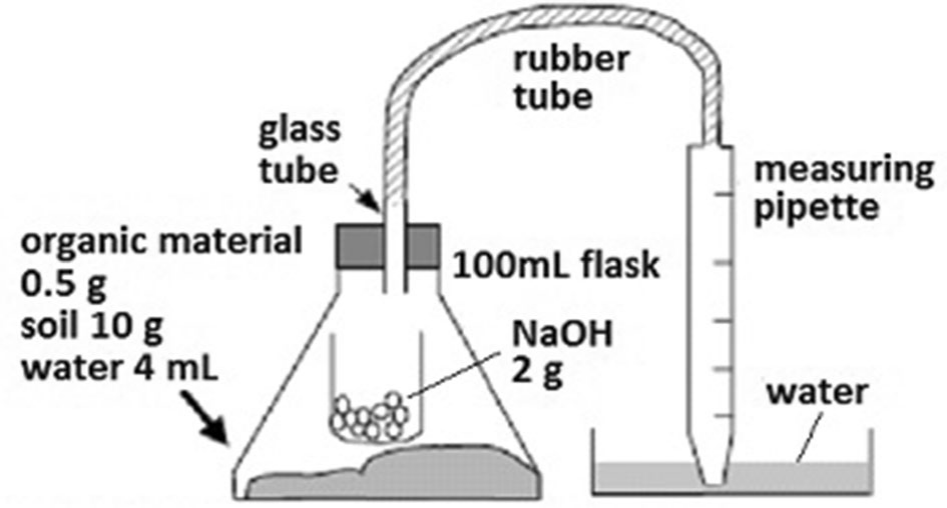
The instrument for the CO_2_ evolution test^41^.

Using a simple respirometric instrument (Fig. 2)^41^, the 10 fast compost samples, 10 test-*kosi* samples, 10 farmer-*kosi* samples, and 10 household wastes samples were incubated at 30 °C for 21 h in the glass flask (Fig. 2), and CO_2_ produced was determined. A 0.5 g air-dried sample was mixed with an air-dried 10 g *arada* soil collected from the mid-altitude dry sub-area and water (60% soil water saturation). A small container that contained a 2 g sodium hydroxide (carbon dioxide absorbent) was placed in the flask. The volume of water sucked by the measuring pipette was measured. From the volume of water measured, the volume of water measured at the control treatment (only the culture soil) was subtracted to obtain the volume of CO_2_ produced. CO_2_ evolved is soluble in aqueous solutions, and the solubility is pH-dependent^9^. Thus, the original pH of each sample was determined. Calcisols^42^ (*Endopetric* *Hypercalcic* *Calcisol*; clay loam) were typical soils in the *arada* fields in the mid-altitude dry sub-area^26^.

### Phytotoxicity bioassay (garden cress germination test)

For the 10 fast compost, 10 test-*kosi*, 10 farmer-*kosi*, 10 household wastes, and 10 *arada* soil samples, phytotoxicity bioassays were conducted.

In a garden cress germination test, the presence of phytotoxic substances is usually determined by a garden cress germination index (GI) that is calculated by the following equation:

$${\text{GI}}\, = \,\left( {{\text{G}}_{{\text{t}}} /{\text{G}}_{{\text{c}}} } \right)\, \times \,({\text{L}}_{{\text{t}}} /{\text{L}}_{{\text{c}}} )$$

where G_t_ = mean germination for treatment, G_c_ = mean germination for distilled water control, L_t_ = mean radicle length for treatment, and L_c_ = mean radicle length for distilled water control. The germination index was rated as follows^43^: 1.0–0.8, no inhibition of plant growth; 0.8–0.6, mild inhibition; 0.6–0.4, strong inhibition; < 0.4, severe inhibition. In this study, GI values for the OF treatments (the OFs mixed with the soil) were compared to those for the soil treatment (the soil only) and a phosphate buffer solution control. The same *arada* soil used in the CO_2_ evolution test was used.

The mean application rate of *kosi* N to *arada* fields in the semi-arid Ethiopian Rift Valley was 67 kg ha^−1^ year^−1^ DM^27^. In this study, consideration was given to mix the OF sample with the culture soil used for incubation at the same application rate of *kosi* N. The weight of the OF sample mixed with the culture soil was determined based on the bulk densities of the *arada* soil at 0–10 cm depth from where the culture soil was collected and total N in the OF sample. Another consideration was given to put the electric conductivity (EC) and pH of the extracts from the OF treatments, the soil treatment, and the control treatment on the same level^44^.

For the OF treatment, after the OF sample mixed with the soil was incubated in a pot at room temperature (22–27 °C) and 60% saturation with water for 1 day, the soil solution was strained through a No. 2 filter (pore size, 8 µm). To the filtrate, 2 mol L^−1^ phosphate buffer solution (1 mol L^−1^ each of the dipotassium hydrogen phosphate and potassium dihydrogen phosphate was contained) was added to adjust the EC of the filtrate to 4.0 dS m^−1^ (the pH becomes 6.8–7.2 then^44^).

The control treatment was prepared by diluting the phosphate buffer solution to make the EC at the same level as the OF extract and soil extract. Two filter papers were placed on a petri dish 6 cm in diameter, on which 3 mL of the OF extract, soil extract, or the phosphate buffer solution corrected (the control) was evenly dropped. Garden cress (*Lepidium sativum*, L*.*) 16 seeds were placed on it, which was kept in the dark at room temperatures (23–27 °C) for 96 h (4 days). Percentage seed germination and radicle lengths were measured for each petri dish to determine the germination index^43,44^.

### Statistical analysis

Multiple comparison tests (the Bonferroni correction) were conducted to detect differences in the mean values of (1) NH_4_^+^ and NO_3_^−^ concentrations and NH_4_^+^:NO_3_^−^ ratio and (2) original pH and the volume of CO_2_ produced between each pair of the four OFs (farmer-*kosi*, test-*kosi*, fast compost, and household wastes). The same statistical tests were conducted for (3) the germination index between each pair of the four OF treatments and the soil treatment. SPSS ver. 20 (IBM) was used for the statistical analysis.

## Results

### Pile temperature changes

In terms of the pile temperature and volume changes, pH, OM, and total N, because no significant differences were observed in the measurement/determination values between 2014 and 2015, only those in 2015 were shown for the fast compost samples.

**Figure 3:**
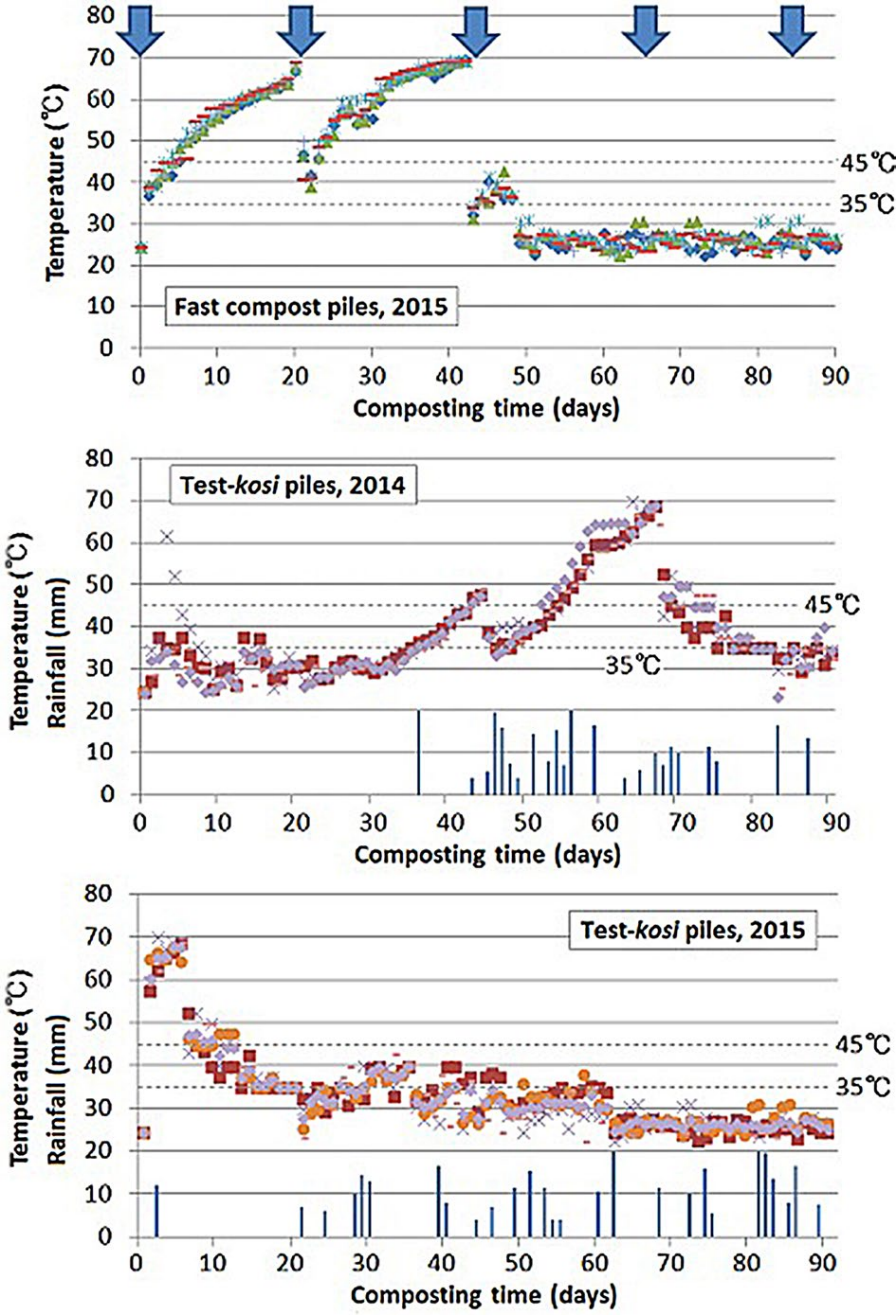
Temperature changes in the test-*kosi* and fast compost piles during the composting process. Arrows indicate watering and pile turning-over.

A remarkable difference in temperature change over the monitoring period was observed between the fast compost and test-*kosi* (2014 and 2015) piles (Fig. 3). The pile temperatures of the fast compost reached the mesophilic temperature range (35–45 °C) at the 2nd day of the monitoring, then rose to the thermophilic temperature-like range (> 45 °C) during the 3rd to 21st days. This cycle of the temperature change was repeated until the third watering and pile turning-over (at the 42nd day), and then the pile temperature became nearly ambient ones after the 50th day.

In contrast, the temperature change in the test-*kosi* piles was largely affected by rainfall conditions. In 2014, no rainfall had been observed until the 36th day (Fig. 3). However, composting began caused by moisture contained inside the feedstock pile, which led some of the test-*kosi* piles to a rise in pile temperatures to the mesophilic or thermophilic range at the 3rd day. After the 20 mm rainfall fell at the 36th day, all the pile temperatures rose to the thermophilic range 5–6 days later. Due to the rain that intermittently fell for 17 days from then, the temperature which had once declined again increased to 68–70 °C. After that, the test-*kosi* pile temperature was within the mesophilic range until the 90th day.

In 2015, due to the 12 mm rain that fell at the 1st day of the monitoring and moisture contained in the test-*kosi* pile, all the piles reached thermophilic temperatures at the 2nd day, then further rose to 68–70 °C 4–5 days later (Fig. 3). After 8–12 days of continuous mesophilic temperatures, the pile temperature decreased to ambient temperatures. After the 3 days of continuous rainfall (at the 27th, 28th, and 29th days), the test-*kosi* pile temperature was within the mesophilic range for 19–30 days. Then, it reached ambient temperatures after the 62nd day.

### Pile volume changes

**Figure 4:**
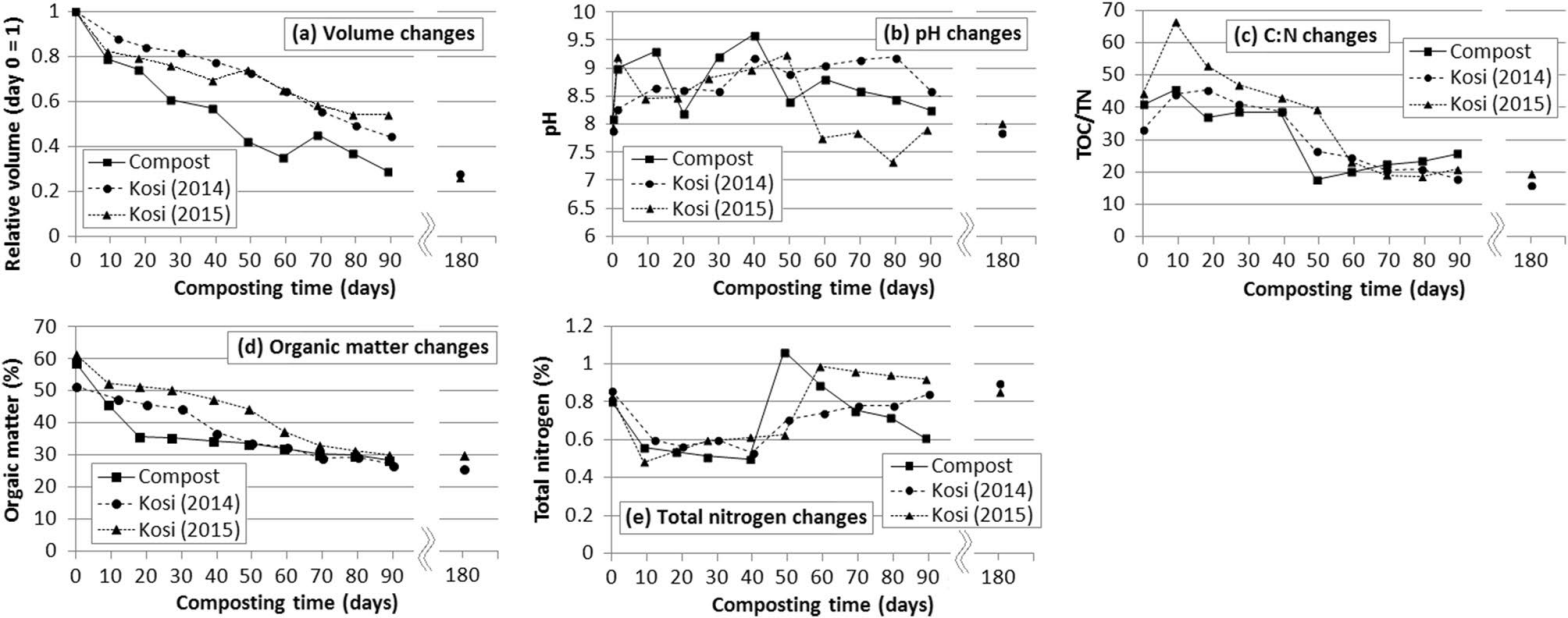
Changes in pile volume (**a**), pH (**b**), C:N ratio (**c**), organic matter contents (**d**), and total N (**e**) of the 2014 and 2015 *kosi* piles and the fast compost pile during the composting process. Mean values were plotted. *C:N ratio* carbon (C):nitrogen (N) ratio, *TOC* total organic carbon, *TN* total nitrogen.

The mean pile volumes for the fast compost samples became 30% (2014) and 29% (2015) of the original, at the 90th day of the monitoring (Fig. 4). Similarly, those for the test-*kosi* piles became 48% (2014) and 54% (2015). The largest volume decreases, 23% for the fast compost pile and 20% for the test-*kosi*, occurred during the first 10 days in 2015. The large volume decreases for the fast compost also occurred during the 20th–30th days and 40th–50th days, while those for the test-*kosi* (2014) occurred during the 50th–60th days (an 11% decrease) and 60th–70th days (a 14% decrease). Thus, large decreases in pile volume for the fast compost occurred immediately after the watering and turning-over, whereas those for the test-*kosi* have occurred shortly after the first high rainfall since the commencement of the monitoring. These pile volume changes corresponded to the periods when the temperatures of the fast compost and test-*kosi* piles increased from the mesophilic range to over 60 °C.

### Pile pH changes

The pH values of both the organic feedstocks and final products of the fast compost and test-*kosi* were alkaline (Fig. 4). The presence of wood ashes in the feedstock may contribute to it^35,45^. For both the fast compost and test-*kosi*, the pile pH rose to approximately 9 twice during the monitoring periods. The pH rises for the fast compost occurred on the pile preparation day (the first watering and turning-over) and immediately after the second and third watering and turning-over. Meanwhile, the first pH rises for both the 2014 and 2015 test-*kosi* occurred after the rainfall events, when the pile temperatures increased from mesophilic to thermophilic ranges. The second pH rises similarly occurred after rainfall events, but those occurred when the pile temperature was in the mesophilic range such as for the 2015 test-*kosi* pile. For both the fast compost and test-*kosi* piles, the second pH rises to ~ 9 continued relatively longer than those of the first pH rises.

### OM content, total N, and C:N ratio changes

The mean OM content of the fast compost largely decreased from 59 to 36% by the 20th day of the monitoring, which corresponded to the first period of the pH rise to ~ 9 (Fig. 4). The 2014 test-*kosi* pile showed a large volume decrease during the 30th–40th days, while the 2015 test-*kosi* pile decreased by the 10th day of monitoring. The occurrence of these large decreases in OM contents corresponded to the period of the first pH rise to ~ 9 that was found after the first rainfall.

Total N contents of every pile largely decreased during the first 10 days of the monitoring, especially for the fast compost that continued to decline until the 40th day (Fig. 4). It was probably due to N losses caused by ammonia volatilisation that occurs during the initial stage of composting^45^. Total N in the fast compost piles largely increased at the 50th day and then gradually decreased. The 50th day was when the fast compost pile temperature changed from the mesophilic to ambient ones. Total N in the test-*kosi* piles largely increased at the 90th day (2014) and 60th day (2015). These corresponded when the periods of the second pH rises to ~ 9 were over, and the pH decreased to 7.7–8.6.

The initial C:N ratios of the fast compost and test-*kosi* were 33–44, almost identical (Fig. 4). The fast compost had a faster-composting process than the test-*kosi*; the C:N ratio of the fast compost became 17 at the 50th day of the monitoring. However, the decrease in total N since then made the ratio 26 on the 90th day. The C:N ratio of the 2014 test-*kosi* became 18 because of the total N rise at the 90th day, while that of the 2015 test-*kosi* became 19 at the 180th day. The final C:N ratios of the 2014 and 2015 test-*kosi* and fast compost did not satisfy a stability/maturity index of C:N ratio, 10–15^11^.

In a composting process, OM decomposition and N losses due to ammonia volatilisation simultaneously occur; however, even after the rate of N loss due to ammonia volatilisation slows, OM degradation continues at a greater rate, resulting in a relative increase of N concentration^46^. However, total N in the fast compost and test-*kosi* (2015) continued to decrease after the 50th day and 60th day, respectively, probably because of NO_3_^−^ leaching or denitrification^47^.

### Weed seeds germination tests

No weed seed germinated was observed among the 80 fast compost, 80 test-*kosi*, and 80 farmer-*kosi* samples, whereas weed seeds germinated were observed in the 7 household wastes samples (Table 1). In the 12 household wastes samples, 15 seeds of *Xanthium spinosum* were found, which were not germinated during the 10 days of the germination test. All the 8 weed species found are generally observed in the crop fields in the semi-arid Ethiopian Rift Valley. In the organic feedstock composing the OFs, weed seeds were originally contained; however, in the composting process associated with an exothermic process reaching ~ 70 °C, these weed seeds can become inactive.

**Table 1:** Weed species and frequencies identified by the weed seed germination test.

Botanical name	Family	Numbers observed			
		Frequency	Petri dishes	Organic fertilisers	Remark
*Setaria pumila (Poir.) Roem. & Schult*	*Poaceae*	3	2	HH. wastes	G
*Sorghum arundinaceum*	*Poaceae*	2	2	HH. wastes	G
*Setaria pumila*	*Poaceae*	1	1	HH. wastes	G
*Commelina benghalensis*	*Commelinaceae*	1	1	HH. wastes	G
*Digitaria abyssinica*	*Poaceae*	1	1	HH. wastes	G
*Solanum nigrum*	*Solanaceae*	1	1	HH. wastes	G
*Cyperus rotundus*	*Cyperaceae*	1	1	HH. wastes	G
*Xanthium spinosum*	*Asteracea*	15	11	HH. wastes	N. G

*HH* wastes, household wastes, *G* germinated, *N.G*. not germinated.

### NO_3_^−^:NH_4_^+^ ratio

The mean NH_4_^+^ contents of the fast compost, test-*kosi*, and farmer-*kosi* samples were significantly lower than that of the household wastes sample (Table 2). The mean NO_3_^−^ contents differed significantly between all the tested OFs, following the order: farmer-*kosi* > test-*kosi* > fast compost > household wastes. Compared to stability/maturity indices established for composts of various origins, < 0.4 mg g^−1^ for NH_4_^+^^48^, > 0.3 mg g^−1^ for NO_3_^–^^49^ and < 1 for NH_4_^+^:NO_3_^−^ ratio^50^, only the farmer-*kosi* and test-*kosi* satisfied all the stability/maturity indices, whereas NO_3_^−^ in the fast compost was lower than the critical limit value of stable/mature compost. The household wastes did not satisfy the stability/maturity indices of either NO_3_^−^ or NH_4_^+^:NO_3_^−^ ratio, probably still being in the composting process.

**Table 2:** (1) NH_4_^+^ and NO_3_^−^ concentrations and NH_4_^+^:NO_3_^−^ ratios; (2) pH and CO_2_ produced; and (3) garden cress germination index.

Organic fertilisers	*n*	(1)			(2)		(3)
		NH_4_^+^ (mg g^−1^)	NO_3_^−^ (mg g^−1^)	NH_4_^+^/NO_3_^−^	pH	CO_2_ produced (mL)	Germination index
Farmer-*kosi*	10	0.011 ± 0.006^**b**^	0.50 ± 0.29^**a**^	0.03 ± 0.02^**b**^	8.16 ± 0.37^**a**^	2.79 ± 1.95^**b**^	3.6 ± 0.8^**b**^
Test-*kosi*	10	0.009 ± 0.004^**b**^	0.34 ± 0.12^**ab**^	0.03 ± 0.02^**b**^	8.05 ± 0.34^**a**^	0.65 ± 1.00^**c**^	6.2 ± 2.2^**a**^
Fast compost	10	0.007 ± 0.001^**b**^	0.23 ± 0.07^**b**^	0.03 ± 0.01^**b**^	8.04 ± 0.38^**a**^	0.64 ± 1.13^**c**^	6.1 ± 0.9^**a**^
Household wastes	10	0.085 ± 0.055^**a**^	0.04 ± 0.07^**c**^	7.58 ± 6.57^**a**^	8.29 ± 0.44^**a**^	8.12 ± 1.35^**a**^	4.1 ± 1.3^**b**^
Only soil (control)	10	–	–	–	–	–	8.7 ± 0.6^**a**^

Different superscript letters indicate statistically significant differences between the organic fertilisers and control (*P* < 0.05).

The NO_3_^−^:NH_4_^+^ ratio of the household wastes had a high standard deviation (Table 2), and the ratio ranged from 0.02, the lowest, to 18.27, the highest. NH_4_^+^ and NO_3_^−^ contents of the household wastes sample that had the NO_3_^−^:NH_4_^+^ ratio of 0.02 were 0.007 mg g^−1^ and 0.248 mg g^−1^, respectively, equivalent to those levels of the farmer-*kosi*. This indicates that household wastes are a mixture of OFs at different phases of the composting process ranging from a composite of fresh organic materials to a composite of those left in the backyards in the long period and passed the similar composting process as a farmer-*kosi*.

### CO_2_ evolution test and phytotoxicity bioassay

The mean pH values of the farmer-*kosi* and household wastes were 8.2 and 8.3, respectively; slightly higher than those of the test-*kosi* and fast compost (Table 2). The volume of CO_2_ produced was higher in the household wastes > farmer-*kosi* > test-*kosi* > fast compost. Significant differences were observed between each pair of the OFs tested except for the relationship between the test-*kosi* and fast compost (*P* < 0.05).

All the garden cress germination index for the OFs tested and the soil were more than 1.0 (Table 2). This proves that any harmful substances that deterred the growth of the garden cress were not contained in the OFs tested or the soil. On the contrary, the garden cress was promoted its growth by the addition of these OFs. The germination index was higher in the culture soil > test-*kosi* > fast compost > household wastes > farmer-*kosi*. The germination indexes of the household wastes and farmer-*kosi* were significantly lower than those of the culture soil, test-*kosi*, and fast compost.

## Discussion

### Composting processes of the fast compost and *kosi*

The combination of favourable conditions, such as easily decomposable OM in the feedstock, moisture (rainfall or watering), temperature (especially mesophilic temperatures^51^), encourages the metabolic activity of a microbial population. Decomposition of easily decomposable OM raises a pile pH to ~ 9^52,53^, leads the emission of ammonia to the peak level^54^, and causes N loss due to ammonia volatilisation^45^. Beck-Friis et al.^54^ found that a total N loss due to ammonia volatilisation, which ranged 24–33% of the initial N content, occurred with thermophilic temperatures and high pH (~ 9). These phenomena were prominently observed during the initial stage of composting when water was supplied to the fast compost pile and when a 10-odd mm of rainwater was added to the moisture contained originally in the organic feedstock of the test-*kosi* in this study. The total amount of ammonia emitted and the duration of the thermophilic temperature phase are related to the initial C:N ratio and the total N content of feedstock^53^. Piles of municipal organic wastes (non-shredded municipal organic waste compost) that had almost the same C:N ratio (C:N = 15 at 50 days after pile establishment), total N content (1.1% at the 50th day), and management practice (turning-over at the 30th, 50th, and 70th days and watering at the 50th day) as the fast compost and test-*kosi* in this study had a similar composting process of this study^35^. It was characterised by a long (95 days) thermophilic temperature phase with a high pH (8.6–8.7) maintained for the whole period of that temperature phase^35^.

For both the fast compost and test-*kosi* piles, the pH rise to ~ 9 was observed twice during the composting process. It was reported that, although the total N contents were identical, the difference in the proportion of easily decomposable OM and hardly decomposable OM in the organic feedstock affected the times of ammonia emission^52,53^. The decomposition of easily decomposable OM occurred in the transition period from the mesophilic to thermophilic temperature ranges, whereas the decomposition of hardly decomposable OM occurred in the transition period from the thermophilic to the subsequent mesophilic temperature ranges^52,53^. The feedstock of fast compost and *kosi* are mixtures of organic materials that have different composition characteristics^12^. For example, tef and barley straws contain a high proportion of acid detergent soluble OM (ADOM^55^) representing readily decomposable OM (OM that is rapidly decomposed within 10–14 days after an application to soil). In contrast, animal dung, such as cattle, donkey, sheep and goat, contains high proportions of mineral and an OM fraction decomposable within 3 months; however, it contains the lowest ADOM among the organic materials^12^. Organic materials with different composition characteristics in both the fast compost and test-*kosi* piles were decomposed in a stepwise manner, which can cause the pH to rise to ~ 9 twice. Decomposition of OM causes the volume reduction of a manure/compost pile^30,31^. These phenomena explain why the large decrease in OM contents for both the fast compost and test-*kosi* piles was always accompanied by watering or rainfall events, large pile volume reductions, and pH rise to ~ 9. Although the composting processes largely differed between the fast compost and test-*kosi*, changes in temperature, volume, pH, and OM and total N contents observed in these composting processes can be explained by the interaction between the microbial activity and OM decomposition.

### Maturity and stability of the organic fertilisers

For both the fast compost and test-*kosi*, the C:N ratio reached the minimum levels when the pile temperatures became ambient, pH decreased, OM contents became the lowest levels, and total N showed the maximum values. The fast compost, 2014 *kosi*, and 2015 *kosi* appeared to reach the maturation phase at the 50th day, after the 90th day, and on the 70th day. Gómez-Brandón et al.^13^ reported that the watering of the mature compost piles rather reduced total N and NO_3_^−^. Watering and turning-over of the fast compost piles were implemented twice even after the piles had reached the maturation phase (at the 50th day). The semi-arid Ethiopian Rift Valley has a rainy season in July and August, which corresponds to the 28th to 89th days of the monitoring period, with 444 mm of the mean rainfall (Welenchiti rainfall gauge, 1992–2013). These watering and rainfall were likely to encourage the NO_3_^−^ leaching and N loss^56^, increasing the mean C:N ratios of the fast compost and test-*kosi* to 26 and 18–19, respectively. Application of cattle manure with a higher C:N ratio poses N immobilisation in soil (e.g., C:N = 15.9^14^; C:N = 15.4^15^). Mukai and Oyanagi^12^ conducted a 42-day incubation test for 10 fast compost and 10 *kosi* samples (the mean N content and C:N ratio of the fast compost sample were 0.89% and 19, respectively). As a result, some *kosi* samples showed a slight immobilisation until the 28th day of incubation, while fast compost showed no noticeable change in net N mineralised, after that, all the samples showed net N mineralised exceeding 0 mg g^−1^. Total N and phosphorus in *kosi* are 1.52% and 0.35%, significantly greater than those of fast compost, 0.89% and 0.24%^12^. The 42-day incubation test found that the mean available N values in 12 weeks (approximately 3 months) after *kosi* and fast compost application to an *arada* soil were estimated to be 1.37 and 1.97 mg g^−1^^12^, being almost the equivalent level. Thus, if mature *kosi* (C:N =  ~ 19)^12^ and mature fast compost (C:N =  ~ 18)^12^ are properly prepared, no large differences will not be found in maturity and stability and short-term N availability between the *kosi* and fast compost.

Weed seeds originally contained in the organic feedstock of the farmer-*kosi*, fast compost, and test-*kosi* became inactive during the composting process. The NH_4_^+^ and NO_3_^−^ contents and NO_3_^−^:NH_4_^+^ ratios of the farmer-*kosi* and test-*kosi* met the standards of mature compost. However, the higher CO_2_ emission for the farmer-*kosi* indicated that a certain level of microbial activity was still maintained in it. The phytotoxicity bioassay probably demonstrated some phytotoxic compounds during the composting process, as reported in Gómez-Brandón et al.^13^. Farmers pile up a fresh organic material on a mature *kosi* pile. Thus, the part of a *kosi* pile that contains newly piled up organic materials may be immature. Considering the 2014 and 2015 *kosi* samples reached the maturity phase on the 90th and 70th days, respectively, if a fresh organic material was added until about 3 months before the time of field application, the whole pile should be mixed so that immature *kosi* is not localised.

*Kosi* preparation does not require much labour, whereas the fast compost preparation requires a great deal of labour for watering and turning-over. The fast compost has probably matured in 50 days. The additional 40 days of work during the remaining monitoring period that included the three times’ watering and pile turning-over was not only the wastes of labour and costs but also led to the decline in compost quality. Ethiopia has diversified agro-ecologies^57^; it can be a local agricultural research centre, which will study an appropriate preparation method of mature fast compost that had an advantage over other local OFs in fast preparation and consistent quality^12^.

Household wastes are a mixture of an OF that has undergone the maturity process similar to *kosi* and a compound of fresh organic materials with different composting processes. Among this, immature household wastes possibly include weed seeds and may produce a severer N immobilisation after its application to soil even more than farmer-*kosi* does. In the 42-day incubation test^12^, all the 10 household wastes samples showed N immobilisation until the 21st–28th day of incubation, and then, the samples were divided into two groups: the one whose N mineralisation prevailed; and another whose net N immobilisation prevailed. In the present practice, a housewife dumps household wastes mainly to *arada* fields that adjoin the homestead. They should stop this practice; they should pile up household wastes on a *kosi* pile made in the backyard instead.

A local agricultural research centre and the administration in semi-arid Ethiopian Rift Valley can also promote technical instructions on the existing *kosi* and household wastes techniques. Besides the aforementioned technical improvements, (1) considering a possible lower rate of seedling emergence and yield reduction due to the net N immobilisation occurred initially for some of the *kosi* samples, it can be recommended to integrate *kosi* into the soil at the ploughing time that is carried out about 1 month before the seed sowing^12^; (2) plant-available N supply from *kosi* is limited in the first *kosi* application year, and *kosi* shows low substitutability for inorganic fertilisers. *Kosi* can be considered to be an OF that contributes to soil cultivation by their medium- or long-term application and to gain crop yields by the increase in total C and N concentrations in a field soil^12^; (3) if a farmer applies *kosi* (7.8 Mg ha^−1^ year^−1^ or 67 kg N ha^−1^) for about 10 years, the farmer is likely to expect 3.0 Mg ha^−1^ of maize in an ordinary rainfall year^27^; and (4) the combination of *kosi* (6.0 Mg ha^−1^ year^−1^) application for about 10 years and 14 kg ha^−1^ year^−1^ inorganic fertiliser N (equivalent to 30 kg ha^−1^ year^−1^ Urea; 30% of the recommended N application) is an alternative option to attain 3.0 Mg ha^−1^ of maize^27^.

Several compost stability tests (self-heating test, monitoring pH and C:N ratio changes, determination of NO_3_^−^:NH_4_^+^ ratio, and CO_2_ evolution test) and compost maturity tests (phytotoxicity bioassays) used in this study are established test methods and have common features of speed, cost-saving, and yet accuracy. Combining it with the simple determination method for N components using RQFlex and the weed germination test in a constant temperature room, the methodology demonstrated in this study can be useful in the field and at a local agricultural research centre close to farmers’ fields.

## Conclusion

Maturity and stability of the local OFs and the fast compost introduced by the administration were evaluated by using several indexes.

Composting of the fast compost began with the first watering to the pile, whereas that of the test-*kosi* began with the first 10-odd mm rainfall. During the active phase of composting, decomposition of easily decomposable OM and ammonia emission occurred with relatively long-lasting thermophilic temperatures and high pH intermittently. The feedstock of *kosi* and fast compost is composed of organic materials that have different decomposition characteristics. By step-by-step decomposition of OM fractions with different OM degradability, phenomena of pH rise to ~ 9 were observed twice during the whole composting process of both the test-*kosi* and fast compost. After that, at the 70th–90th days and 50th day of the monitoring when the C:N ratios were at the lowest levels, the test-*kosi* and fast compost appeared to reach the maturity phase, respectively.

Weed seeds originally contained in the feedstock of the *kosi* and fast compost became inactive during the composting process. However, a certain level of active microbial was still maintained in the farmer-*kosi* samples, indicating the probable presence of some phytotoxic compounds. High C:N ratios of *kosi* and fast compost produce N immobilisation shortly after application to the field. Considering the period required for *kosi* maturity (70–90 days), farmers should mix mature *kosi* and immature *kosi* in a pile first and then apply it to a field about 1 month before the seeding. A challenge is to determine how researchers can apply the enhanced predictive understanding of the quality of OFs to assist farmers in managing their organic resources^58^. For this, a local agricultural research centre needs to accumulate several standard indexes for maturity and stability of local OFs.

## Supplementary Information

Supplementary Information

## References

[1] Van Ittersum MK, van Bussel LGJ, Wolf J, Grassini P, van Wart J, Guilpart N (2016). Can sub-Saharan Africa feed itself?. PNAS.

[2] Kimetu JM, Lehmann J, Ngoze SO, Mugendi DN, Kinyangi J, Riha SJ, Verchot L, Recha JW, Pell AN (2008). Reversibility of soil productivity decline with organic matter of differing quality along a degradation gradient. Ecosystems.

[3] Yimer F, Abdelkadir A (2010). Soil property changes following conversion of acacia woodland into grazing and farmlands in the Rift Valley area of Ethiopia. Land Degrad. Dev..

[4] Morris M, Kelly VA, Kopicki RJ, Byerlee D (2007). Fertiliser Use in African Agriculture. Lessons Learned and Good Practice Guidelines.

[5] Mekonnen, A. & Köhlin, G. *Biomass Fuel Consumption and Dung Use as Manure: Evidence from Rural Households in the Amhara Region of Ethiopia* (Environment for Development, 2008).

[6] Endale K (2011). Fertilizer Consumption and Agricultural Productivity in Ethiopia.

[7] Minten, B., Tamru, S., Engida, E., Kuma, T. *Ethiopia’s Value Chains on the Move: The Case of teff. ETHIOPIA Strategy Support Program (ESSP) II* (ESSP and IFPRI, 2013).

[8] Vanlauwe B, Chianu J, Giller KE, Merckx R, Mokwunye U, Pypers P (2010). Integrated soil fertility management: Operational definition and consequences for application and dissemination. Outlook Agric..

[9] Gómez RB, Vázquez-Lima F, Sánchez-Ferrer A (2006). The use of respiration indices in the composting process: A review. Waste Manag. Res..

[10] Wu L, Ma LQ, Martinez GA (2000). Comparison of methods for evaluating stability and maturity of biosolids compost. J. Environ. Qual..

[11] Chefetz B, Hatcher PG, Hadr Y, Chen Y (1996). Chemical and biological characterization of organic matter during composting of municipal solid waste. J. Environ. Qual..

[12] Mukai S, Oyanagi W (2019). Decomposition characteristics of indigenous organic fertilisers and introduced quick compost and their short-term nitrogen availability in the semi-arid Ethiopian Rift Valley. Sci. Rep..

[13] Gómez-Brandón M, Lazcano C, Domínguez J (2008). The evaluation of stability and maturity during the composting of cattle manure. Chemosphere.

[14] Castellanos JZ, Pratt PF (1981). Mineralization of manure nitrogen-correlation with laboratory indexes. Soil Sci. Soc. Am. J..

[15] Beauchamp EG (1986). Availability of nitrogen from three manures to corn in the field. Can. J. Soil Sci..

[16] Materechera SA, Modiakgotla LN (2006). Cattle manure increases soil weed population and species diversity in a semi-arid environment. South Afr. J. Plant Soil.

[17] Palm CA, Rowland AP, Cadisch G, Giller KE (1997). Chemical characterization of plant quality for decomposition. Driven by Nature: Plant Litter Quality and Decomposition.

[18] Gachengo CN, Vanlauwe B, Palm CA, Cadisch G, Delve RJ, Probert ME (2004). Chemical characterisation of a standard set of organic materials. Modelling Nutrient Management in Tropical Cropping Systems.

[19] Lupwayi NZ, Girma M, Haque I (2000). Plant nutrient contents of cattle manures from small-scale farms and experimental stations in the Ethiopian highlands. Agr. Ecosyst. Environ..

[20] Onduru DD, Snijders P, Muchena FN, Wouters B, Jager DA, Gachimbi L, Gachini GN (2008). Manure and soil fertility management in sub-humid and semi-arid farming systems of sub-Saharan Africa: Experiences from Kenya. Int. J. Agric. Res..

[21] Tittonell P, Rufino MC, Janssen BH, Giller KE (2009). Carbon and nutrient losses during manure storage under traditional and improved practices in smallholder crop-livestock systems—evidence from Kenya. Plant Soil.

[22] Lekasi JK, Tanner JC, Kimani SK, Harris PJC (2003). Cattle manure quality in Maragua District, Central Kenya: Effect of management practices and development of simple methods of assessment. Agr. Ecosyst. Environ..

[23] Markewich HA, Pell AN, Mbugua DM, Cherney DJR, van Es HM, Lehmann J, Robertson JB (2010). Effects of storage methods on chemical composition of manure and manure decomposition in soil in small-scale Kenyan systems. Agr. Ecosyst. Environ..

[24] Ofosu-Budua GK, Hogarhb JN, Fobile JN, Quayea A, Dansoc SKA, Carbood D (2010). Harmonizing procedures for the evaluation of compost maturity in two compost types in Ghana. Resour. Conserv. Recycl..

[25] Stewart ZP, Pierzynski GM, Middendorf BJ, Prasad PVV (2020). Approaches to improve soil fertility in sub-Saharan Africa. J. Exp. Bot..

[26] Mukai S (2018). Assessment of long-term soil dynamics at manured fields with field measurement and interviews: A case study in the semi-arid Ethiopian Rift Valley. Agroecolo. Sustain. Food Syst..

[27] Mukai S (2018). Historical role of manure application and its influence on soil nutrients and maize productivity in the semi-arid Ethiopian Rift Valley. Nutr. Cycl. Agroecosyst..

[28] MoARD (2005). Community Based Participatory Watershed Development: A Guideline.

[29] Rynk R, van de Kamp M, Wilson GB (1992). On-Farm Composting Handbook.

[30] Zheng GD, Chen TB (2004). Dynamic of lead specialization in sewage sludge composting. J. Water Sci. Technol..

[31] Seal A, Bera R, Chatterjee AK, Dolui AK (2012). Evaluation of a new composting method in terms of its biodegradation pathway and assessment of the compost quality, maturity and stability. Arch. Agron. Soil Sci..

[32] Wichuk KM, McCartney D (2010). Compost stability and maturity evaluation—a literature review. Can. J. Civ. Eng..

[33] Gao M, Liang F, Yu A, Li B, Yang L (2010). Evaluation of stability and maturity during forced-aeration composting of chicken manure and sawdust at different C/N ratios. Chemosphere.

[34] Sánchez-Monedero MA, Roig A, Paredes C, Bernal MP (2001). Nitrogen transformation during organic waste composting by the Rutgers system and its effects on pH, EC and maturity of the composting mixtures. Biores. Technol..

[35] Tognetti C, Mazzarino MJ, Laos F (2007). Improving the quality of municipal organic waste compost. Bioresour. Technol..

[36] Temesgen M, Rockstrom J, Savenije HHG, Hoogmoed WB, Alemu D (2008). Determinants of tillage frequency among smallholder farmers in two semi-arid areas in Ethiopia. Phys. Chem. Earth.

[37] Mkhabela TS, Materechera SA (2003). Factors influencing the utilization of cattle and chicken manure for soil fertility management by emergent farmers in the moist Midlands of KwaZulu-Natal Province, South Africa. Nutrient Cycl. Agroecosyst..

[38] Jiménez EI, García VP (1992). Relationships between organic carbon and total organic matter in municipal solid wastes and city refuse composts. Biores. Technol..

[39] Ando Y, Oyanagi W, Moriyama N (2004). A simple method for the determination of nutrients content in organic matter using the small reflection photometer. Jpn. Soc. Soil Sci. Plant Nutr..

[40] Tanahashi T, Yano H, Itou H, Oyanagi W (2010). Magnesium ammonium phosphate in cattle and swine manure composts and an extraction method for its evaluation. Jpn. Soc. Soil Sci. Plant Nutr..

[41] Oyanagi W, Ando Y (2007). A method of estimating decomposable organic matter by measuring oxygen consumption. Bull. Niigata Anim. Husband. Exp. Stat..

[42] FAO (2006). World Reference Base for Soil Resources 2006: A Framework for International Classification, Correlation and Communication.

[43] Zucconi F, Pera A, Forte M, de Bertoldi M (1981). Evaluating toxicity of immature compost. BioCycle.

[44] Fujiwara T, Hara M, Murakami K (2004). Proposal for germination test of animal waste compost that reflects the rate of application and reduces the effect of EC and pH of water extract. Jpn. J. Soil Sci. Plant Nutr..

[45] Mengistu T, Gebrekidan H, Kibret K, Woldetsadik K, Shimelis B, Yadav H (2017). Comparative effectiveness of different composting methods on the stabilization, maturation and sanitization of municipal organic solid wastes and dried faecal sludge mixtures. Environ. Syst. Res..

[46] Dias BO, Silva CA, Higashikawa FS, Roig A, Sanchez-Monedero MA (2010). Use of biochar as bulking agent for the composting of poultry manure: Effect on organic matter degradation and humification. Bioresour. Technol..

[47] Rufino MC, Rowe EC, Delve RJ, Giller KE (2006). Nitrogen cycling efficiencies through resource-poor African crop–livestock systems. Agric. Ecosyst. Environ..

[48] Zucconi F, de Bertoldi M, de Bertoldi M, Ferranti MP, Hermite PL, Zucconi F (1987). Compost specification for the production and characterization of compost from municipal solid waste. Compost: Production, Quality and Use.

[49] Forster JC, Zech W, Wurdinger E (1993). Comparison of chemical and microbio-logical methods for the characterization of the maturity of composts from contrasting sources. Biol. Fertil. Soils.

[50] Brewer LJ, Sullivan DM (2003). Maturity and stability evaluation of composted yard trimmings. Compost Sci. Util..

[51] Sundberg C, Smårs S, Jӧnsson H (2004). Low pH as an inhibiting factor in the transition from mesophilic to thermophilic phase in composting. Biores. Technol..

[52] Beck-Friis B, Smars Y, Jonsson S, Eklind Y, Kirchmann H (2003). Composting of source-separated household organics at different oxygen levels: Gaining an understanding of the emission dynamics. Compost. Sci. Util..

[53] Beck-Friis B, Smars S, Jonsson H, Kirchmann H (2001). Gaseous emissions of carbon dioxide, ammonia and nitrous oxide from organic household waste in a compost reactor under different temperature regimes. J. Agric. Eng. Res..

[54] Pagans E, Barrena R, Font X, Sánchez A (2006). Ammonia emissions from the composting of different organic wastes. Dependency on process temperature. Chemosphere.

[55] Oyanagi W, Tanahashi T, Muramatsu K, Kobashi Y (2010). Utility of acid detergent soluble organism as index of decomposing organic materials easily. Jpn. J. Soil Sci. Plant Nutr..

[56] Tulema B, Aune JB, Breland TA (2007). Availability of organic nutrient sources and their effects on yield and nutrient recovery of tef [*Eragrostis tef* (Zucc.) Trotter] and on soil properties. J. Plant Nutr. Soil Sci..

[57] Suryabhagavan KV (2017). GIS-based climate variability and drought characterization in Ethiopia over three decades. Weather Clim. Extremes.

[58] Giller KE (2000). Translating science into action for agricultural development in the tropics: An example from decomposition studies. Appl. Soil. Ecol..

