# Supplementary material for: Evaluation on maturity and stability of organic fertilisers in semi-arid Ethiopian Rift Valley
Source: Sci Rep. 2021 Feb 17;11:4035. doi: 10.1038/s41598-021-83611-4 (PMC7890062; doi:10.1038/s41598-021-83611-4)
Supplement: Supplementary file 1 — Supplementary Information [file 41598_2021_83611_MOESM1_ESM.docx]

**Supplementary Information for**

**Evaluation on maturity and stability of organic fertilisers in semi-arid Ethiopian Rift Valley**

Shiro Mukai^1^ & Wataru Oyanagi^2^

^1^ Independent scholar, 1-19-7 Omorinishi, Ota-ku, Tokyo 143-0015, Japan. ^2^Niigata Agricultural Research Institute Livestock Research Center, Niigata 955-0143, Japan.

**Table S1.** Determination of nitrogen components using RQ-flex and comparison with the standard laboratory determination methods. **^a^** When a 100 g manure sample was digested with 500 mL of 100 g L^-1^ potassium chloride solution, the determined values of the sample that contained more than 1 g kg^-1^ NH_4_^+^ showed a tendency of being lower than those values measured by the standard methods. This was a similar finding obtained from Van Kessel & Reeves (Ref.**^55^**). Thus, the concentration of the potassium chloride solution was doubled. For the extract whose concentration value exceeded a confidential level (60 mg L^-1^ NH_4_^+^), it was further diluted and determined again. **^b^** A 100 g manure sample was digested with 1,000 mL of 100 g L^-1^ potassium chloride solution. The extract was diluted as necessary. **^c^** A test paper for a high-concentration solution (20－180 mg L^-1^ NH_4_^+^) was basically used; however, for the sample whose concentration value was too low, a test paper for a low-concentration solution (0.2－7.0 mg L^-1^ NH_4_^+^) was used. Most of the dairy slurry samples were determined using the test paper for a high-concentration solution with pretreatment of a five-fold dilution.

| Sources | Samples | RQ-flex test paper | Extraction methods | Standard methods | Equation and coefficient of determination |
| --- | --- | --- | --- | --- | --- |
| Total N**^1^** | Manure (cattle, swine, chicken; *n* = 44), other organic materials (*n* = 48) | Ammonium test (measuring range of 0.2–7.0 mg L^-1^ NH_4_^+^) | Sulphuric acid and hydrogen peroxide digestion (X) | Wet Kjeldahl method (Y) | Y = 0.830 X (R^2^ = 0.949) |
| NH_4_^+^ **^2^** | Dairy manure (*n* = 107) | Ammonium test (20–180 mg L^-1^ NH_4_^+^) | Stream distillation (X) | Stream distillation ( Y) | Y = 0.84 X + 0.34 (R^2^ = 0.91) |
| NH_4_^+^ **^3^** | Dairy manure (*n* = 41) | Ammonium test (20–180 mg L^-1^ NH_4_^+^)**^a^** | 100 g L^-1^ potassium chloride (X) | Stream distillation ( Y) | Y = 1.199 X + 0.084 (R^2^ = 0.973) |
|  | Dairy manure (*n* = 36) | Ammonium test (0.2－7.0 mg L^-1^ NH_4_^+^)**^b^** | 100 g L^-1^ potassium chloride (X) | Stream distillation ( Y) | Y = 1.158 X + 0.003 (R^2^ = 0.963) |
|  | Dairy slurry (*n* = 86) | Ammonium test (20–180 mg L^-1^ NH_4_^+^)**^c^** | 100 g L^-1^ potassium chloride (X) | Stream distillation ( Y) | Y = 0.974 X + 0.089 (R^2^ = 0.912) |
| NO_3_^-^ **^1^** | Manure (cattle, swine, chicken; *n* = 44), other organic materials (*n* = 48) | Nitrate test (5－225 mg L^-1^ NO_3_^-^) | 0.1 M sulphuric acid (X) | Stream distillation ( Y) | Y = 0.993 X (R^2^ = 0.995) |

**References**

1. Ando, Y., Oyanagi, W. & Moriyama, N. A simple method for the determination of nutrients content in organic matter using the small reflection photometer. *Japanese Society of Soil Science and Plant Nutrition* **75(5)**, 605–608 (2004) (in Japanese).
2. Van Kessel, J. S. & Reeves III, J. B. On-farm quick tests for estimating nitrogen in dairy manure. *J. Dairy Sci*. **83**, 1829–1836 (2000).
3. Matsumoto, T. Studies on the fertility management of timothy sward based on use of dairy cattle manures evaluated as available nutrients, *Bulletin of Hokkaido Reserch Organization Agricultural Experiment Stations* **121**, 1–61 (2008) (in Japanese).
